# Effect of Moringa leaf flavonoids on the production performance, immune system, and rumen fermentation of dairy cows

**DOI:** 10.1002/vms3.993

**Published:** 2022-12-10

**Authors:** Ji Liu, Yan Wang, Ling Liu, Guangming Ma, Yonggen Zhang, Jian Ren

**Affiliations:** ^1^ College of Food and Bioengineering Qiqihar University Qiqihar China; ^2^ College of Animal Science and Technology Northeast Agricultural University Harbin China

**Keywords:** dairy cow, *Moringa oleifera* leaf flavonoids, nutrient digestibility, ruminal fermentation

## Abstract

**Background:**

Unreasonable use of antibiotics in animals is a major concern and will remain so, thus affecting people's health. However, the application of plant extracts can better solve this problem.

**Objectives:**

The purpose of this study was to study the effect of Moringa leaf flavonoids on the production performance, immunity, and rumen fermentation of dairy cows.

**Methods:**

Nine Holstein multiparous cows (average weight: 550 kg; days of lactation: 150 ± 6 days) were used in the experiment, using a 3 × 3 Latin square design. Cows were divided into three groups, each of which was supplemented with 0, 50, or 100 mg/body weight (BW) *Moringa oleifera* leaf flavonoids. Each experimental period consisted of three periods of 21 days, and the prefeeding period lasted 15 days.

**Results:**

Our results indicated that supplementation with Moringa leaf flavonoids increased the protein content and decreased the number of somatic cells in milk; had little effect on the biochemical indices of blood, the rumen fermentation, and serum biochemical indicators; and improved the activity of antioxidant enzymes, the antioxidant capacity, and immunity.

**Conclusions:**

Addition of 50 mg/BW Moringa leaf flavonoids to cow enhanced the antioxidant and immunity capacity in dairy cows but did not affect physiological levels of common biochemical parameters in blood or fermentation parameters in rumen.

## Graphical abstract

In this study, we found that adding 50mg/BW moringa leaf flavonoids increased the protein content in milk, reduced the number of somatic cells, and had no effect on milk production, milk fat, total solids, and other indicators. At the same time, it improves the antioxidant capacity and immunity of dairy cows and does not affect blood physiological and biochemical indicators and rumen fermentation parameters.

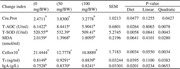

## 1 INTRODUCTION

Antibiotics are commonly used as supplement in ruminant feeds to improve production performance. However, the inappropriate use of antibiotics in feeds can lead to drug resistance in animals and drug residues in food, thus posing threats to human health (Byarugaba, 2004). The nontherapeutic use of antibiotics in animal feeds have been banned in the European Union and many other countries, due to the concerns of the spread of antibiotic resistance from animal products to consumers (Rojo et al., 2015). The use of some antibiotics has been banned worldwide. Therefore, there is a need to evaluate the potential of natural antibacterial agents (e.g., plant extracts) to improve the production capacity of animals (Kamel, 2006).

Flavonoids are polyphenolic compounds commonly found in plants as secondary metabolites. Flavonoids have proven abilities in enhancing immunity in animals, through their anti‐pathogenic, anti‐oxidation, and anti‐inflammatory functions. Flavonoids can affect rumen metabolism and rumen microbial fermentation (Linville et al. 2018) and promote the circulation of blood in dairy cows, thereby enhancing their metabolism, promoting the absorption of nutrients, and increasing milk production (Zhan et al., 2017). Flavonoids also have a weak estrogenic effect, which regulates the secretion of growth hormone and promotes breast development (Liu et al., 2020). Flavonoids can promote nitrogen metabolism in the rumen of dairy cows and can reduce methane production (Leake & Rankin, 1990).

Moringa is rich in nutrients and biologically active compounds, and thus has great potential to be used as a supplement in livestock feeds. The leaves, seeds, and bark of Moringa can be readily consumed by cows, sheep, goats, pigs, chickens, and rabbits (Radványi et al., 2013). Consumption of Moringa has been proven to improve the health, growth performance, milk production, and meat quality of livestock (Nardone & Valfrè, 1999). Moringa contains various flavonoids in the leaves, roots, flowers, and seed coats, and the contents of flavonoids vary depending the geographic origins of Moringa. The most common flavonoids in Moringa leaves are kaempferol, quercetin, isorhamnetin, and apigenin (Milugo et al., 2013).

So far, there have been few reports of the effects of Moringa leaves extracts rich in flavonoids on dairy cows. The aim of this study was to evaluate the effects of Moringa leaves flavonoids extract on the production performance, immune responses, and ruminal fermentation of dairy cows.

## 2 MATERIALS AND METHODS

### 2.1 Materials

Nine Holstein cows from Wonderson pasture in Harbin were used in the experiment. The cows were in milk for 150 ± 6 days, with average body weight (BW) of 550 ± 25 kg. The experiment was conducted in a 3 × 3 Latin square design, consisting of three 21‐day experimental periods, with 15 days for adaptation. Cows were divided into three groups and fed a total mixed ration (TMR) (Table 1) supplemented with 0, 50, and 100 mg/BW Moringa leaf flavonoids. Moringa leaf flavonoids (purity, 50%) were produced by Shaanxi Quanao‐Engineering Co. Ltd. (Xi′an, China) (See Table 2, 3, 4, 5, 6).

**TABLE 1:** Ingredients of the total mixed ration (TMR)

Ingredients	Content (%)
Corn	26
Bran	5
Cottonseed meal	5
Soybean meal	6
DDGS	4
Rumen bypass fat	1
Puffed soybeans	2
Cottonseed	2
Salt	0.5
Beet meal	2
Premix	0.5
Alfalfa	10
Soybean hulls	2
Silage	25
*Leymus chinensis*	9

*Note*: The premix per kilogram diet contained 2900 IU of vitamin A, 31,500 IU of vitamin D, 40 IU of vitamin E, 120 mg of iron, 15 mg of copper, 40 mg of zinc, 0.3 mg of iodine, 0.1 mg of selenium, and 0.10 mg of cobalt.

Abbreviation: DDGS, Distillers Dried Grains with Solubles.

**TABLE 2:** Effect of Moringa leaf flavonoids on the milk yield and milk composition of dairy cows

Milk components	0 mg/BW	50 mg/BW	100 mg/BW	SEM	*p*‐Value		
					Diet	Linear	Quadratic
CP	2.4711^b^	3.8300^a^	3.2778^b^	1.0213	0.0477	0.1255	0.0427
Fat	4.0700	3.9089	3.8889	0.1721	0.3108	0.1679	0.5236
Lactose	5.4411	5.3711	5.3711	0.07595	0.6253	0.4078	0.6298
TS	12.6467	13.6911	13.1011	1.1220	0.1597	0.3892	0.0862
Cells × 10^4^	21.4444^a^	12.7778^c^	16.8889^b^	1.7183	0.0034	0.0550	0.0034
UN mg/dl	30.0348	30.0348	29.2416	3.0742	0.9299	0.7455	0.8511
Milk yield (kg)	34.32	34.42	34.09	1.22	0.9125	0.800	0.78

*Note*: In the same row, values with different letters indicate significant differences (*p* > 0.05), and the same or no letter indicates no significant differences (*p* < 0.05).

Abbreviations: BW, body weight; CP, crude protein; SEM, standard error of the mean; TS, total solids; UN, urea nitrogen.

**TABLE 3:** Effect of Moringa leaf flavonoids on blood biochemical indicators

Blood biochemical indicators	0 mg/BW	50 mg/BW	100 mg/BW	SEM	*p*‐Value		
					Diet	Linear	Quadratic
TP (g/L)	65.9136	64.6517	64.6597	1.8735	0.8204	0.5930	0.7538
ALB (g/L)	30.3236	29.8730	29.3573	0.5265	0.4427	0.2068	0.9602
GLB (g/L)	35.6256	34.7121	35.2408	1.6146	0.8833	0.8368	0.6566
HDL (mmol/L)	3.7229	3.7419	3.7626	0.1643	0.9492	0.7496	0.9938
GLU (mmol/L)	3.2179	3.3597	3.2989	0.1445	0.4554	0.4748	0.3052
LDH (U/L)	915.31	979.88	916.83	61.1840	0.2305	0.9707	0.0923

Abbreviations: ALB, albumin; BW, body weight; GLB, globulin; GLU, glucose; HDL, high‐density lipoprotein; LDH, low‐density lipoprotein; SEM, standard error of the mean; TP, total protein.

**TABLE 4:** Effect of Moringa flavonoids on the rumen fermentation index

Volatile acid	MLF			SEM	*p*‐Value		
	0 mg/BW	50 mg/BW	100 mg/BW		Diet	Linear	Quadratic
Acetate mmol/L	90.72	82.95	83.31	5.88	0.34	0.217	0.42
Propinate mmol/L	34.39	31.05	30.66	1.88	0.32	0.17	0.53
Butyrate mmol/L	17.82	17.37	17.34	1.41	0.96	0.81	0.9
Vaterrate mmol/L	1.99	2.04	1.98	0.95	0.96	0.96	0.75
Isobutyrate mmol/L	0.611	0.6	0.62	0.06	0.98	0.91	0.87
Isovalerate mmol/L	2.04	2.33	2.13	0.27	0.68	0.79	0.41
TVFA mmol/L	147.76	136.51	135.77	0.41	0.41	0.24	0.54

Abbreviations: BW, body weight; MLF, Moringa leaves Flavonids; SEM, standard error of the mean; TVFA, total volatile acid fatty acid.

**TABLE 5:** Effect of Moringa flavonoids on the antioxidant indexes of dairy cows

Antioxidants	0 mg/BW	50 mg/BW	100 mg/BW	SEM	*p*‐Value		
					Diet	Linear	Quadratic
MDA (nmol/ml)	2.0159^a^	1.3968^b^	1.8095^b^	0.2196	0.0641	0.4101	0.0280
GSH‐Px (U/ml)	15.3597	16.0189	16.0168	1.9200	0.7250	0.4945	0.6898
T‐AOC (U/ml)	6.1422^b^	8.4415^a^	5.9041^b^	0.6801	0.0264	0.8065	0.0078
T‐SOD (U/ml)	520.55^ab^	532.39^a^	509.41^b^	5.2745	0.0058	0.0841	0.0043
CAT (U/ml)	2.3687	2.2935	2.3286	0.3021	0.9807	0.9175	0.8693

*Note*: In the same row, values with different letters indicate significant differences (p>0.05), and the same or no letter indicates no significant differences (*p* < 0.05).

Abbreviations: BW, body weight; CAT, catalase; GSH‐Px, glutathione peroxidase; MDA, malondialdehyde; SEM, standard error of the mean; T‐AOC, total antioxidant capacity; T‐SOD, total superoxide dismutase.

**TABLE 6:** Effect of Moringa flavonoids on the immune performance indexes of dairy cows

Immune performance indexes	0 mg/BW	50 mg/BW	100 mg/BW	SEM	*p*‐Value		
					Diet	Linear	Quadratic
T_3_ (ng/ml)	0.8149^b^	0.9291^a^	0.8839^b^	0.03244	0.0395	0.1100	0.0383
T_4_ (ng/ml)	76.9780	82.1682	77.1260	2.0907	0.0920	0.9536	0.0315
PRL (ng/ml)	243.68	256.09	255.00	13.0871	0.3065	0.2072	0.3782
IgA (g/L)	0.7528^b^	0.8370^a^	0.8241^a^	0.03301	0.0201	0.0234	0.0653
IgG (g/L)	11.4310	11.0116	11.5069	0.4696	0.5564	0.8773	0.2922
IgM (g/L)	2.8509	2.6024	2.8393	0.1436	0.2016	0.9388	0.0789

*Note*: In the same row, values with different letters indicate significant differences (*p* > 0.05), and the same or no letter indicates no significant differences (*p* < 0.05).

Abbreviations: BW, body weight; PRL, prolactin; SEM, standard error of the mean.

## 3 METHODS

### 3.1 Testing of milk samples

The milk samples were stored at 4°C and then submitted to Heilongjiang DHI Testing Center for analysis with a multifunctional dairy analyzer. The parameters measured included milk fat rate, milk total solids, lactose rate, milk protein rate, milk urea nitrogen, and somatic cell count.

### 3.2 Blood biochemical test

On the 20th day of each phase of the test, blood was collected from the tail vein with a coagulation vacuum tube (containing inert separating gel) before and 2 h after ingestion. The blood was allowed to stand for 1 h at room temperature, followed by centrifugation to separate the serum, which was then aliquoted and stored at −20°C until further analysis.

The main blood biochemical indicators were total protein, albumin (ALB), globulin (GLB), glucose (GLU), high‐density lipoprotein (HDL), and low‐density lipoprotein (LDL). The main hormones were prolactin (PRL), triiodothyronine (T_3_), and tetraiodothyronine (T_4_).

### 3.3 Volatile acid determination

The temperature of the inlet and detector was 220°C. The oven temperature scheme started with initial temperature at 120°C for 3 min, and then increased to 180°C at 10°C/min. The carrier gas was high‐purity nitrogen. The port pressure was maintained at 90 kpa; the hydrogen flow rate was 40 ml/min, the airflow rate was 400 ml/min, and the makeup flow rate was 45 ml/min.

### 3.4 Determination of antioxidant and immune indicators

The immunoglobulin content (IgG, IgM, and IgA) was determined by radioimmunoassay, which was completed by the Beijing Huaying Institute of Biotechnology. Antioxidant indicators, total superoxide dismutase (T‐SOD), total antioxidant capacity kit (T‐AOC), glutathione peroxidase (GSH‐Px), catalase (CAT), and malondialdehyde (MDA) were analyzed using the test kit (Nanjing Jiancheng Institute of Biology) according to the manufacturer's instructions.

### 3.5 RESULTS

#### 3.5.1 Effect of Moringa leaf flavonoids on dairy cow performance, blood biochemistry, and rumen fermentation

##### 3.5.1.1 Effect of Moringa leaf flavonoids on dairy cow performance

The protein content in the milk was increased by the diet supplemented with 50 mg/BW Moringa leaf flavonoids compared to the control (*p* < 0.05), which was the lowest in this latter diet among all three diets administered. There were no significant changes in milk fat, lactose, and total solids among the three diets. The number of somatic cells in the diet supplemented with 50 mg/BW Moringa leaf flavonoids decreased significantly and was significantly lower than the control, suggesting that the addition of 50 mg/BW Moringa leaf flavonoids significantly reduced the number of somatic cells. The above results show that the addition of an appropriate amount of Moringa leaf flavonoids to the diet can increase the protein content in milk and significantly reduce the number of somatic cells.

##### 3.5.1.2 Effect of Moringa leaf flavonoids on blood biochemical indicators

The blood total protein, ALB, GLB, low‐density lipoprotein (LDH), and GLU content increased with the increase of the amount of Moringa leaf flavonoids with some noticeable variations. The content of HDL increased as the amount of the Moringa leaf flavonoids increased, and HDL was the highest in the diet supplemented with 100 mg/BW. However, no significant differences were observed among treatments. The above data indicate that Moringa leaf flavonoids had no significant effect on blood biochemical indicators.

##### 3.5.1.3 Effect of Moringa leaf flavonoids on the volatile acids of milk

The content of acetic acid, propionic acid, and butyric acid in milk was lower in the treatments supplementation with Moringa leaf flavonoids than the control. The content of valeric acid, isobutyric acid, and isovaleric acid was the highest in the treatment with 50 mg/BW Moringa leaf flavonoids, but no significant differences were observed among diets. Based on the above results, the addition of Moringa leaf flavonoids in the diet did not show significant effects on rumen fermentation parameters.

##### 3.5.1.4 Effect of Moringa leaf flavonoids on antioxidant indexes

The addition of 50 mg/BW Moringa leaf flavonoids significantly reduced the content of MDA, significantly increased the content of T‐AOC and T‐SOD, and had no significant effect on the content of GSH‐PX and CAT.

##### 3.5.1.5 Effect of Moringa leaf flavonoids on immune performance indicators

The IgA content was significantly higher under supplementation with 50 mg/BW Moringa leaf flavonoids than under supplementation with 0 mg/BW and 100 mg/BW Moringa leaf flavonoids. The T_3_ content was significantly higher under supplementation with 50 mg/BW Moringa leaf flavonoids than under supplementation with 0 mg/BW and 100 mg/BW Moringa leaf flavonoids. Moringa leaves had no significant effect on the flavonoids PRL and T_4_ and other blood biochemical indicators. The above results indicate that Moringa leaf flavonoids affect serum biochemical indicators.

## 4 DISCUSSION

The addition of appropriate amount of Moringa leaf flavonoids did not increase milk production in dairy cows. Some studies have shown that the addition of flavonoids in the form of grape pomace powder, green tea, and turmeric extracts does not increase milk production (Williamson et al., 2005). The results of our study are consistent with these findings. However, previous work has shown that the addition of propolis flavone as a feed additive has no effect on the number of somatic cells in the milk of dairy cows, which is inconsistent with the results of our study showing that the addition of Moringa leaf flavonoids decreases the number of somatic cells. This inconsistency can be explained by the different sources, types, and structures of flavonoids used (Zicker & Wedekind, 2005). Elevated somatic cells in milk indicate poor breast health and milk quality, which is a major problem in modern animal husbandry. Cows with elevated somatic cells produce less milk than healthy cows, which results in major economic losses to the dairy industry. Approximately 70% of subclinical mastitis is related to a temporary or permanent decrease in milk production, which mainly stems from inflammatory damage to breast tissue (Eckersall et al., 2006). In general, nutritional factors play a key role in enhancing resistance to breast infections, and excessive amounts of micronutrients with effective antioxidant and immune‐enhancing properties added to the diet such as plant extracts have been reported to enhance breast health and reduce the somatic cell count (Scaletti et al., 2003). Milk production is lower in cows with higher initial somatic cell levels. Previous studies have shown that breast infections may significantly reduce milk production (Tikofsky et al., 2003). Previous work indicates that damage to secretory tissue and fibrous tissue can lead to breast infection, which causes a temporary or permanent decrease in milk production (Lauzon et al., 2006). The biologically active compounds in Moringa leaf flavonoids discovered in this study show anti‐inflammatory activity and reduce the number of somatic cells.

No effect of supplementation with Moringa leaf flavonoids on the concentration of HDL and LDL was observed. Hosoda et al. (2006) also observed no difference in the concentrations of HDL and LDL. Changes in blood biochemical indicators can provide insight into animal health, nutrient metabolism, and production performance. The level of serum total protein, the GLB content, and ALB content reflect the body's absorption of protein and protein metabolism. In this study, Moringa leaf flavonoids showed no effect on serum total protein, including GLB, ALB, HDL, GLU, and LDL.

Rumens are unique digestive organs of ruminants that contain a large number of microorganisms, which can obtain nutrients from feed through fermentation processes. Regulation of the rumen can improve the utilization rate of feed and promote the production performance of ruminants. The volatile acids produced by rumen fermentation of dairy cows are important energy sources for dairy cows, among which acetic acid, propionic acid, and butyric acid are the three main volatile acids accounting for more than 80% of the total volatile acids (Foiklang & Toburan, 2011). Volatile fatty acids are the main product of carbohydrate degradation in the rumen, the main energy source for ruminants, and the main raw material for synthetic milk fat.

Xi et al. (2007) found that the addition of vegetable oils significantly reduced total volatile fatty acid (TVFA) production. Using in vitro culture methods and hay as the fermentation substrate, Vasta et al. (2008) showed that cinnamon oil has no significant effect on the concentration of volatile acids and the ratio of acetic acid and propionic acid.

Kwekkeboom et al. (1993) studied the effect of capsaicin on the rumen fermentation of lactating dairy cows and showed that these plant essential oils had no effect on TVFAs, their composition, and ammonia nitrogen. The findings of our study were similar to their results, as Moringa leaf flavonoids did not affect the rumen fermentation index.

Free radicals play an important role in immunity and signal transduction, but excessive free radicals can lead to the lipid peroxidation of cell membranes (Turner et al., 2004). Endogenous antioxidant enzymes, including SOD, CAT, and GSH‐Px, neutralize oxidative stress, which is the main form of intracellular defence (Manor et al., 1999). The antioxidant effect is achieved by the conversion of oxygen‐free radicals into weakly oxidized forms (Kamel, 2005). Supplementation of flavonoids can improve antioxidant capacity, improve non‐specific immunity, and reduce oxidative stress by increasing SOD and GSH‐Px activity while reducing the concentration of MDA. The underlying mechanism involves flavonoids acting as reducing agents and hydrogen donors to neutralize oxygen‐free radicals and remove hydrogen peroxide and superoxide ions (Kahkonen, 1999).

In this study, the addition of Moringa leaf flavonoids increased the activity of T‐SOD (*p* < 0.01) and T‐AOC (*p* < 0.05) in plasma and reduced the activity of MDA. These results are consistent with the observations that flavonoids induced antioxidant enzyme activity in rat liver and kidney. In addition, Campbell et al. (2013) observed that the flavonoids of Tartary buckwheat in the diet increased the antioxidant capacity of sheep plasma and liver. Similarly, Vasta and Luciano (2011) reported that adding tannins to the diet of lambs can improve the overall antioxidant status parameters of muscles. The possible reason for these findings is that flavonoids may selectively induce the expression of antioxidant enzyme genes by activating the nuclear factor E2‐related factor Nrf2. Yeh and Yen (2005) found that the mRNA abundance of SOD, GSH‐Px, and CAT in the liver of rats with phenolic compounds added was higher than that of Nrf2 protein in the control group and may play a key role in the activation of antioxidant genes induced by phenolic compounds.

Therefore, Moringa leaf flavonoids are antioxidants with diverse effects worthy of further study. The results of our study suggest that Moringa leaf flavonoids enhance the body's immune function by stimulating endogenous antioxidant enzyme activity and protecting the body from oxidative stress.

The immune response is closely related to animal health (Ingvartsen & Moyes, 2013). Determination of the serum immunoglobulin concentration is one of the most commonly used methods for evaluating immunity. The addition of 50 mg/BW Moringa leaf flavonoids significantly increased the content of IgA, and changes in IgG and IgM were not pronounced. Measurement of immune indicators revealed that Moringa leaf flavonoids have a regulatory effect on the immune system of cows and improve the body's immune function. Immunoglobulin is a globulin that can be used as an antibody and has a chemical structure similar to antibodies. Immunoglobulins play a role in antigen‐specific binding and regulation of the immune response.

The addition of 50 mg/BW Moringa leaf flavonoids significantly increased the content of T3, and changes in PRL and T4 were not pronounced.

Moringa leaf flavonoids can increase the concentration of immunoglobulin in the plasma of dairy cows. Moringa leaf flavonoids have been shown to play a role in immune function. Based on the results of previous research, we speculate that Moringa leaf flavonoids stimulate mitochondrial adenosine triphosphate production, which may be mediated by active oxidants and enhance cellular/mitochondrial antioxidant status. However, the specific mechanism by which Moringa leaf flavonoids enhance immune function requires further study (Leonard et al., 1984). The addition of Astragalus extract in the diet can reduce the plasma T_3_ level, which may lead to a decrease in the basal metabolic rate of heat‐stressed broilers and increase the energy required for broiler growth. These findings differ from the results of this experiment. This may be explained by the difference in the number of stomachs between ruminants and other animals. There was no difference in the concentration of T_4_ among the groups in this experiment. Cooke et al. (2011) showed that T_3_ in dairy cows is higher in diets containing tea pollen; however, tea pollen has no effect on the T_4_ concentration (Taraghijou et al., 2012). In this study, T_3_ increased significantly in dairy cows fed with 50 mg/BW Moringa flavonoids, which is consistent with the results of previous studies.

## 5 CONCLUSIONS

In conclusion, the addition of 50 mg/BW Moringa leaf flavonoids to cow diet increased the protein content in milk, but had no effect on milk yield/day, mild fat, total solids, or other quality traits. Additionally, Moringa leaf flavonoids enhanced the antioxidant and immunity capacity in dairy cows but did not affect physiological levels of common biochemical parameters in blood or fermentation parameters in rumen.

## AUTHOR CONTRIBUTIONS

*Conceptualization, resources, writing—original draft, and writing—review and editing*: Ji Liu. *Software and supervision*: Yan Wang. *Data curation and supervision*: Ling Liu. *Formal analysis and methodology*: Guangming Ma. *Funding acquisition*: Yonggen Zhang. *Data curation and funding acquisition*: Jian Ren.

## CONFLICTS OF INTEREST

The authors declare no conflict of interest.

## ETHICS STATEMENT

All procedures used in this study were approved by the Institutional Animal Care and Use Committee of Northeast Agricultural University.

### PEER REVIEW

The peer review history for this article is available at https://publons.com/publon/10.1002/vms3.993.

## Data Availability

All data generated or analyzed during this study are included in this article.
